# Responses of Intestinal Antioxidant Capacity, Morphology, Barrier Function, Immunity, and Microbial Diversity to Chlorogenic Acid in Late-Peak Laying Hens

**DOI:** 10.3390/ani14202957

**Published:** 2024-10-14

**Authors:** Yue Sun, Zhuang Li, Ming Yan, Haitong Zhao, Zhengxing He, Mingkun Zhu

**Affiliations:** 1 Jiangsu Key Laboratory of Sericultural Biology and Animal Biotechnology, School of Biotechnology, Jiangsu University of Science and Technology, Zhenjiang 212100, China; sunyue2036@163.com (Y.S.); 19549147738@163.com (Z.L.); 17669478840@163.com (M.Y.); haitongz0323@163.com (H.Z.); 2 Key Laboratory of Silkworm and Mulberry Genetic Improvement, Ministry of Agriculture and Rural Affairs, The Sericultural Research Institute, Chinese Academy of Agricultural Sciences, Zhenjiang 212100, China; 3 Dantu Borough Animal Disease Prevention and Control Center, Zhenjiang 212100, China; zxinghe2024@163.com

**Keywords:** chlorogenic acid, barrier function, immune response, cecal microbiota, laying hen

## Abstract

**Simple Summary:**

The intestines of layers during the late laying phase usually exhibit lipid metabolism disorders, concurrent with impaired energy production and antioxidant capacity. Therefore, it is necessary to explore feed additives that can enhance production performance through preserving gut barrier integrity and balancing the microbiota. Chlorogenic acid (CGA) exhibits potent pharmacological effects, including antioxidant, anti-inflammatory, antibacterial, antiviral, and lipid metabolism-regulating properties. However, the impact of CGA on late-peak laying hens remains to be further studied. In this study, we investigated the effects of CGA on gut antioxidant status, morphology, barrier function, immunity, and cecal microbiota of late-peak laying hens. Our study provides evidence that CGA treatment can enhance intestinal antioxidant status, improve intestinal morphology, strengthen intestinal barrier and immune function, and promote beneficial gut microbiota growth in laying hens during the late-peak laying period.

**Abstract:**

This study examined the influence of chlorogenic acid (CGA) on gut antioxidant status, morphology, barrier function, immunity, and cecal microbiota in late-peak laying hens. A total of 240 Hy-Line Brown hens, aged 43 weeks, were randomly assigned to four groups, the basal diet +0, 400, 600, and 800 mg/kg CGA, for 12 weeks. The results revealed that CGA significantly reduced ileal H_2_O_2_ and malondialdehyde levels; increased duodenal height, ileal villus height, and villus height-to-crypt depth ratio; while decreasing jejunal crypt depth. The 600 and 800 mg/kg CGA significantly upregulated the duodenal, jejunal, and ileal ZO-1 and occludin gene expression; increased IgG levels in serum and ileum; and upregulated ileal IgA gene expression. The 600 mg/kg CGA significantly upregulated CD3D and CD4 gene expression, while downregulating IL-1β gene expression in duodenum, jejunum, and ileum. Moreover, CGA changed the gut microbiota structure. The SCFA-producing bacteria *unclassified_f__Peptostreptococcaceae*, *unclassified_f_Oscillospiraceae*, *Pseudoflavonifractor*, *Lachnospiraceae_FCS020_group*, *Oscillospira*, *Elusimicrobium*, *Eubacterium_ventriosum_group*, *Intestinimonas*, and *norank_f_Coriobacteriales_Incertae_Sedis* were significantly enriched in the 400, 600, and/or 800 mg/kg CGA groups. The bacteria *Lactobacillus*, *Bacillus*, and *Akkermansia* were significantly enriched in the 600 mg/kg CGA group. Conclusively, dietary CGA (600–800 mg/kg) improved intestinal antioxidant status, morphology, barrier and immune function, and beneficial microbiota growth in late-peak laying hens.

## 1. Introduction

It is well known that gut health is crucial for the overall well-being of the body, which not only significantly affects feed intake and effective absorption of nutrients [1,2], but also plays a critical role in maintaining immunological balance [3]. Gut microbiota is essential for nutrient metabolism, intestinal development, and the formation of a fully functioning immune system [4,5]. Wang et al. indicated that the fecal flora of high-producing layers could improve the laying rate of low-producing layers by regulating the relative abundance of gut flora [6]. Additionally, enhancing intestinal mucosal barrier functions has also been shown to positively impact the production performance of laying hens [7]. The intestines of layers during the late laying phase usually exhibit lipid metabolism disorders, concurrent with impaired energy production and antioxidant capacity [8]. Therefore, it is necessary to explore feed additives that can enhance production performance by preserving gut barrier integrity and balancing the microbiota.

Chlorogenic acid (CGA), a phenylpropanol compound derived from plant aerobic respiration, is a prominent component in numerous Chinese herbs like Lonicera japonica Thunb flowers and Eucommia ulmodies and Chrysanthemum indicum L leaves [9,10]. CGA exhibits potent pharmacological effects, including antioxidation, anti-inflammatory, antibacterial, antiviral, anti-tumor, anti-diabetes, and regulation of lipid metabolism [11,12,13]. In light of the risks posed to human health by the improper use of antibiotics, as well as the regulatory limitations on antibiotic use in livestock production, the exploration of safe, natural plant alternatives mirroring medicinal attributes for livestock production stands is becoming a current focal point in animal husbandry research [14].

Research has demonstrated that utilizing probiotics can enhance the health of the intestine and promote a balanced intestinal flora [15]. CGA has gained significant attention for its prebiotic properties in recent years. When CGA is hydrolyzed by specific esterases in the rat intestine into caffeic acid and quinic acid, both its prototype and hydrolyzed forms can be absorbed by the gastrointestinal tract (GI) [16]. Around 15–32% of consumed CGA undergoes hydrolysis to caffeic acid in the cecum, where it is further metabolized into small molecular weight phenolic acids [16,17]. Due to its extensive activity against pathogenic microorganisms, CGA has been recognized as a potential naturally occurring antibiotic and antiviral alternative [18]. In vitro antibacterial test shows that CGA has inhibitory activity against Gram-negative bacteria such as *E. coli* and *Shigella dysenteriae* [19,20]. Multiple studies have found the impacts of CGA on gastrointestinal health and gut microbiota regulation, providing a possible pathway to improve gut health and growth performance. For example, CGA addition improves intestinal morphological integrity, enhances serum and intestinal antioxidant potential, reduces ileal MDA content, upregulates ZO-1, occludin, and claudin-1 gene expression, and increases the *Bifidobacterium* and *Lactobacillus* abundance in weaned piglets [21]. In addition, in broilers and young hens fed CGA diet, it was found that the decline in intestinal function and disturbance in gut flora caused by heat stress, high stocking density, etc., can be significantly alleviated [22,23].

We hypothesized that dietary supplementation with CGA could beneficially enhance the overall health of late-peak laying hens by affecting intestinal morphology and modifying the composition of the microbial community. Hence, our purpose was to assess the influence of CGA on the gut barrier, immune response, antioxidant status, and microbial diversity in laying hens. The research will establish a theoretical foundation for the potential utilization of CGA in practical production.

## 2. Materials and Methods

### 2.1. Animals and Treatment Schedule

A total of 240 43-week-old Hy-Line Brown hens with similar body weight (1.99 ± 0.20 kg) and laying rates (90.00 ± 1.08%) were obtained from a commercial chicken farm in Zhenjiang, China. They were randomly assigned into four groups, each with six replicates of ten hens (2 hens per cage). The layers were then fed a basal diet added with 0, 400, 600, and 800 mg/kg CGA (powder, 98% CGA, Lvyouran Biotech (Xi’an, China) Co., Ltd., Xi’an, China). The different doses of CGA powder were thoroughly mixed with the basal diet. The dietary CGA supplementation dosage was based on the report by Liu et al. [24]. The experiment lasted for 14 weeks including a 2-week pre-feeding period (fed with basal diet) and a 12-week experimental period. They were housed in a naturally ventilated chicken house, with the temperature controlled between 23–26 °C, humidity maintained at 65–75%, and a lighting period of 16 h/D. The hens had free access to feed and water. The basal diet was designed following the NRC (1994) guidelines to fulfill the nutritional needs of laying hens (Table 1).

### 2.2. Sample Collections

Following the completion of the experiment, 12 hens from each group (2 hens per replicate) were chosen at random to undergo 12 h fasting (free access to water). Blood samples were then collected via venipuncture of the wing vein and centrifuged at 3000 rpm for 10 min to isolate serum for immunoassay analysis. Finally, the chicken was euthanized by CO_2_ inhalation, and intestinal samples, including the duodenum, jejunum, and ileum (the middle of each part), were dissected into three parts. One part was sampled in 4% paraformaldehyde for histomorphologic analysis; the other two parts were stored at −80 °C for antioxidant, immune, and molecular biological analysis. The cecum (seven samples were selected from each group, with at least one sample in each replicate) was gently isolated and ligated with sterile surgical thread. It was then quickly transferred to a sterile workstation, where the cecal contents were aseptically scraped for subsequent intestinal microbiological analysis.

### 2.3. Intestinal Antioxidant Capacity and Immune Factor Assays

In the preparation, each ileal tissue sample weighing 0.1 g was homogenized in precooled PBS at a ratio of nine parts PBS to one part tissue. The supernatant was obtained following centrifugation at 2500 g for 10 min at 4 °C to evaluate the antioxidant ability. The total superoxide dismutase (T-SOD, No. A001-1), glutathione peroxidase (GSH-Px, No. A005-1) and catalase (CAT, No. A007-1) activities, and the malondialdehyde (MDA, No. A003-1) and hydrogen peroxide (H_2_O_2_, No. A064-1) contents were measured using commercial kits (Nanjing Jiancheng Bioengineering Institute, Nanjing, China), following the manufacturer’s instructions. The immunoglobulin G (IgG) contents in serum and ileal tissue were assessed using an IgG (chicken) ELISA test kit (No. BY-EC660107, BoYan Biotechnology Co., Ltd. Nanjing, China) following the manufacturer’s instructions. The absorbance was measured using a microplate reader (BioTek, Shoreline, WA, USA).

### 2.4. Histomorphology Studies

Analysis of intestinal histomorphology followed the method described by Chen et al. [25]. Briefly, the intestinal samples were immersed in four percent paraformaldehyde for a minimum of 24 h, followed by embedding in paraffin. The samples were then cut into 5 μm thick sections with a paraffin microtome (Leica RM2016, Wetzlar, Germany). The tissue sections were dewaxed with xylene and dehydrated using a gradient of ethanol solutions. The sections were imaged utilizing an OLYMPUS optical microscope (Tokyo, Japan) after being stained with hematoxylin and eosin. Ten random intact villi from each slice sample were selected for analysis. Villi height (VH), crypt depth (CD), and the VH-to-CD ratio (VH/CD) were then calculated.

### 2.5. Microbial Diversity Measurement

The procedures for measuring microbial diversity refer to a previous study reported by Ding et al. [26]. Microbial genomic DNA was extracted from cecal content samples using genomic DNA kits from Tiangen Biotech Co., Ltd. (Beijing, China) following the manufacturer’s instructions. The concentration of DNA was determined with a NanoDrop2000, and its quality was assessed through 2% agarose gel electrophoresis. The V3 and V4 hypervariable regions of the microbial 16S rDNA gene were amplified using the primers 341F “CCTAYGGGRBGCASCAG” and 806R “GGACTACNNGGGTATCTAAT”. The equimolar mix of purified amplicons was sequenced using paired-end sequencing (2 × 300) on an Illumina MiSeq platform (San Diego, CA, USA) following the manufacturer’s instructions. The Majorbio Cloud Platform (http://www.majorbio.com) was utilized for species composition analysis, diversity analysis, and differential analysis.

### 2.6. Total RNA Extraction and qRT-PCR Analysis

Total RNA was extracted from liver tissues using Trizol reagent and complementary DNA (cDNA) was synthesized using a cDNA Reverse Transcription kit (Takara RR047A, Dalian, China). Quantitative real-time PCR (qRT-PCR) was run on the CFX96 Touch RT-PCR detection system (Bio-Rad, Hercules, CA, USA) using TB Green Premix Ex Taq (Takara RR420A). The target gene expression level was normalized to the level of β-actin. The 2^−ΔΔCt^ method was used to calculate the relative expression of each gene [27]. The specific primer sequences are listed in Table 2.

### 2.7. Data Analysis

Data were presented as the mean ± SEM. SPSS 19.0 was used for statistical significance analysis. Using one-way ANOVA followed by Tukey’s post-hoc test, group comparisons were conducted. A *p*-value < 0.05 indicated statistical significance. The differences in microbiome data were assessed using the Wilcoxon rank sum test.

## 3. Results

### 3.1. Effects of CGA on Intestinal Antioxidant Capacity

As shown in Table 3, supplementation of 400, 600, and 800 mg/kg CGA notably reduced the ileal MDA and H_2_O_2_ levels of laying hens compared to control (*p* < 0.05). Treatment with 600 mg/kg of CGA significantly increased the ileal GSH-Px activity (*p* < 0.05).

### 3.2. Effects of CGA on Intestinal Morphology

**Figure 1:**
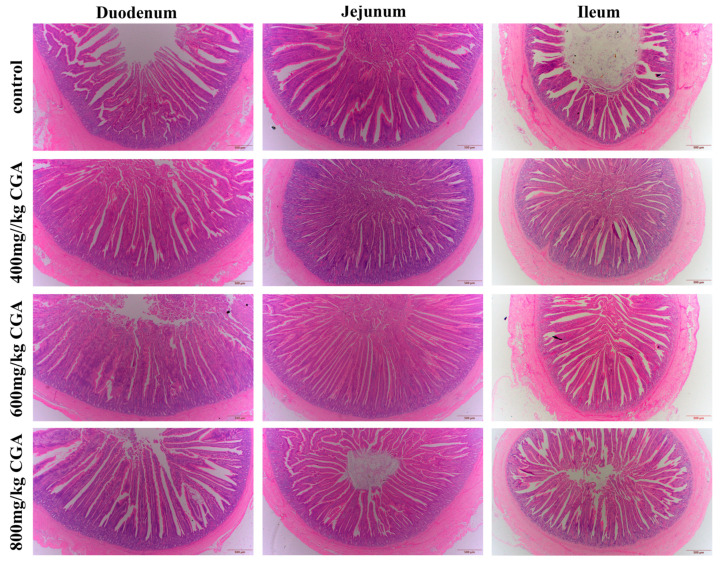
Effects of CGA supplementation on the intestinal morphology in laying hens. Scale bar = 500 μm. CGA: chlorogenic acid.

As illustrated in Figure 1 and Table 4, a significant increase was observed in ileal VH and VH/CD in all CGA groups compared to the control (*p* < 0.05). Additionally, the duodenal VH presented a significant increase in all CGA groups, with a significant increase in the duodenal VH/CD in the 800 mg/kg CGA group (*p* < 0.05). In the jejunum, CD significantly decreased in all CGA supplement groups, and the VH and VH/CD increased significantly in the 600 mg/kg CGA group compared to the control (*p* < 0.05). 

### 3.3. Effects of CGA on the Intestinal Barrier

**Figure 2:**
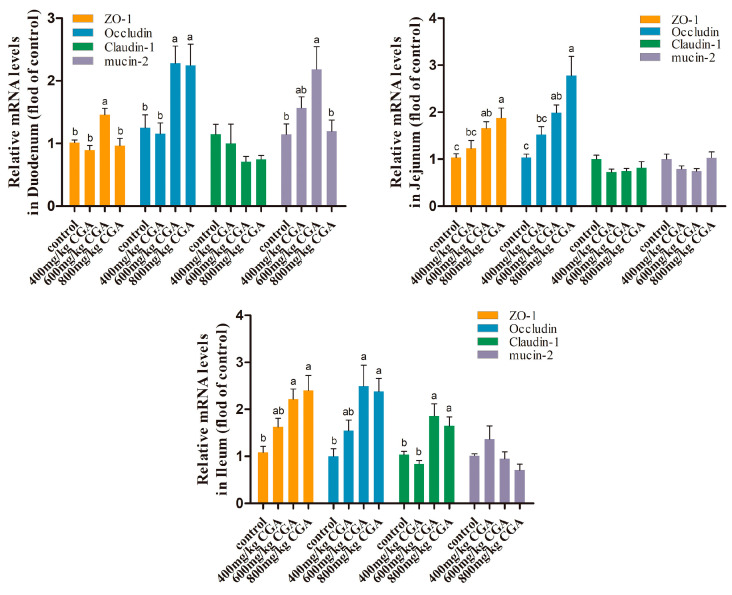
Effects of CGA on intestinal barrier function-related gene expression in laying hens. Data are expressed as the mean value accompanied by SEM (*n* = 12). ^a–c^ Bar charts annotated with distinct superscript letters indicate statistically significant differences (*p* < 0.05). Abbreviation: ZO-1: zonula occludens-1.

As shown in Figure 2, supplementation of 600 and 800 mg/kg CGA significantly upregulated ileal ZO-1, claudin-1, and occludin gene expressions of laying hens (*p* < 0.05). The jejunal ZO-1 and occludin gene expressions were significantly upregulated by 600 and 800 mg/kg CGA (*p* < 0.05). Meanwhile, in the duodenum, the expression of ZO-1, occludin, and mucin-2 genes in the 600 and/or 800 mg/kg CGA group were significantly upregulated compared with the control (*p* < 0.05).

### 3.4. Effects of CGA on Aryl Hydrocarbon Receptor (AHR) Pathway in Intestine

**Figure 3:**
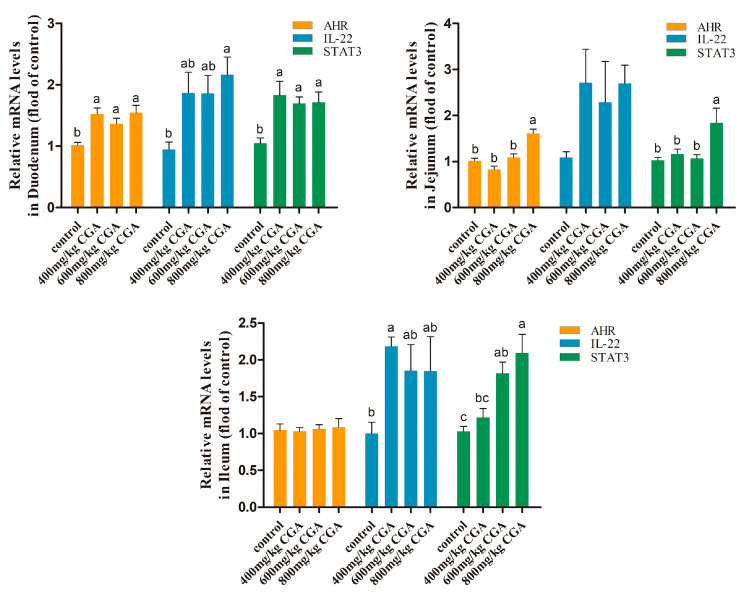
Effects of CGA on AHR/IL-22/STAT3 signaling pathway in the intestinal tract of laying hens. Data are expressed as the mean value accompanied by SEM (*n* = 12). ^a–c^ Bar charts annotated with distinct superscript letters indicate statistically significant differences (*p* < 0.05). Abbreviation: IL-22: interleukin-22; AHR: aryl hydrocarbon receptor; STAT3: signal transducer and activator of transcription 3.

In this experiment, we evaluated the impact of CGA on the AHR signal. The results are presented in Figure 3. Compared with the control, 400, 600, and 800 mg/kg CGA treatment significantly upregulated the expression of AHR, interleukin-22 (IL-22), and signal transducer and activator of transcription 3 (STAT3) genes in the duodenum of laying hens (*p* < 0.05). In the jejunum, AHR, IL-22, and STAT3 genes were significantly upregulated only in the 800 mg/kg CGA group (*p* < 0.05). Treatment with 600 and 800 mg/kg of CGA significantly upregulated the STAT3 gene expression in the ileum (*p* < 0.05).

### 3.5. Effects of CGA on Immunity

**Figure 4:**
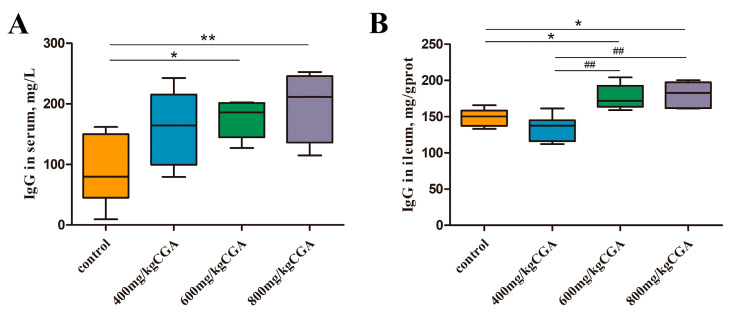
Effects of CGA on immune factor levels in serum (**A**) and ileum (**B**) of laying hens. Data are expressed as the mean value accompanied by SEM (*n* = 12). * *p* < 0.05 and ** *p* < 0.01 indicate a significant difference compared to the control group; ## *p* < 0.01 indicates a significant difference compared to the group of 400 mg/kg CGA.

Additionally, we evaluated the immune response of layers at the systemic and ileal levels, and the data are shown in Figure 4. Compared with the control, the IgG concentrations in serum and ileum of layer exposure to 600 and 800mg/kg CGA were increased significantly (*p* < 0.05).

### 3.6. Effects of CGA on Expression of Intestinal Immune-Related Genes

**Figure 5:**
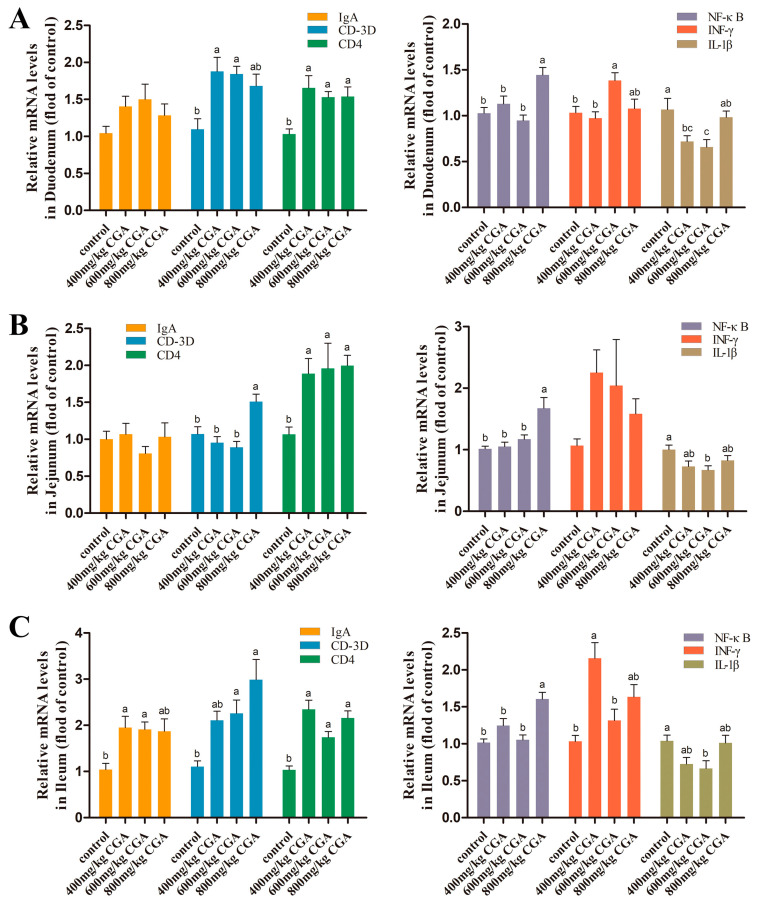
Effects of CGA on intestinal immune-related gene expression in laying hens: (**A**) duodenum; (**B**) jejunum; (**C**) ileum. Data are expressed as the mean value accompanied by SEM (*n* = 12). ^a–c^ Bar charts annotated with distinct superscript letters indicate statistically significant differences (*p* < 0.05). Abbreviation: IgA: immunoglobulin A; IL-1β: interleukin-1 beta; INF-γ: interferon-gamma; NF-κB: nuclear factor-kappa B.

As shown in Figure 5. Compared with the control, CGA treatment significantly upregulated the CD-3D and CD4 expression in the duodenum, jejunum, and ileum (*p* < 0.05). The IgA expression was only upregulated significantly in the ileum of laying hen exposure to CGA (*p* < 0.05). Supplementation of 800 mg/kg CGA significantly upregulated NF-κB expression in three intestinal segments (*p* < 0.05). Supplementation of 600 mg/kg CGA significantly downregulated IL-1β expression in three intestinal segments, and significantly increased IFN-γ expression in duodenum (*p* < 0.05).

### 3.7. Effects of CGA on Cecal Microbiota

**Figure 6:**
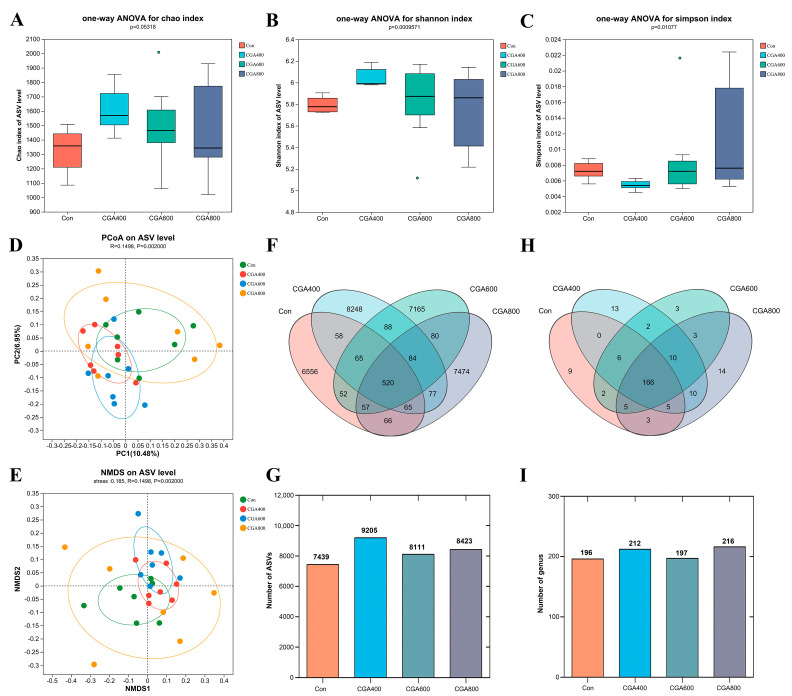
Effects of CGA on microbial diversity in the cecum of laying hens: (**A**–**C**) Alpha diversity index analysis boxplot. (**D**) PCoA and (**E**) NMDS analysis of the cecum microbiota based on the bary_surtis metric. Venn diagrams of ASV (**F**,**G**) and genus (**H**,**I**) distribution in different groups. *n* = 7 hens per group (at least 1 hen per replicate). Con: control; CGA 400: 400 mg/kg chlorogenic acid; PCoA: principal coordinate analysis; NMDS: non-metric multidimensional scaling; ASV: amplicon sequence variant.

As shown in Figure 6, the alpha diversity among different groups is not significant (*p* > 0.05) (Figure 6A–C). Analysis using principal coordinate analysis (PCoA) and non-metric multidimensional scaling (NMDS) indicated significant differences between the 400 and 600 mg/kg CGA groups and the control group (*p* < 0.05) (Figure 6D,E). The ASV number increased in CGA-exposed groups (Figure 6F,G). At the genus level, there are 166 common genera between groups. For control, 400, 600, and 800 mg/kg CGA groups, each group had 9, 13, 3, and 14 distinct genera, respectively (Figure 6H,I).

**Figure 7:**
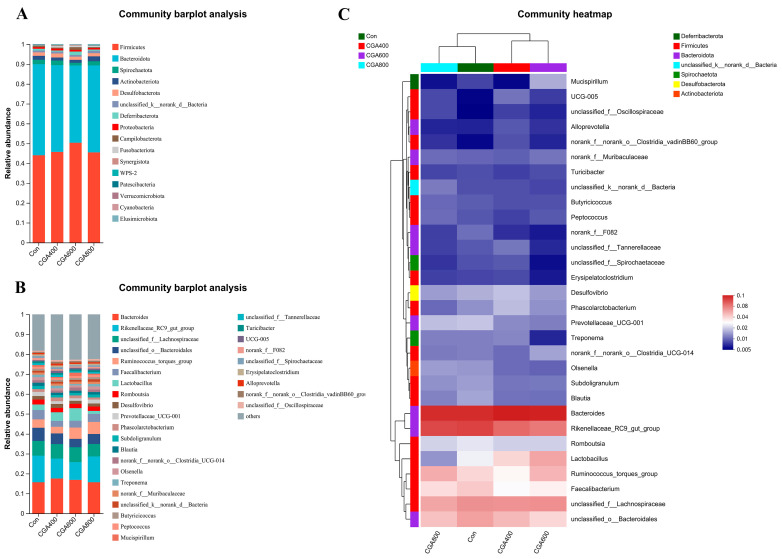
Bar graph illustrating the relative abundance of species at both the phylum (**A**) and genus levels (**B**). (**C**) Heatmap of species relative abundance clustering. *n* = 7 hens per group (at least 1 hen per replicate). Con: control group; CGA 400: 400 mg/kg CGA group; CGA 600: 600 mg/kg CGA group; CGA 800: 800 mg/kg CGA group.

To determine changes in specific bacterial groups after dietary supplementation with CGA, we analyzed the community composition of all taxa at phyla and genus levels. The results are shown in Figure 7. At the phylum level, the predominant bacteria in each group were *Firmicutes* and *Bacteroidetes*. The dominant genera identified included *Bacteroides*, *Rikenellaceae_RC9_gut_group*, *unclassified_f_Lachnospiraceae*, *unclassidied_o_Bacteroidales*, *Ruminococcus_torques_group*, *Faecalibacterium*, and *Lactobacillus* (Figure 7A,B). The heat map, which was generated by selecting the 30 most abundant genera and clustering them at both the species and sample levels, clearly depicts significant variations in abundance among the groups (Figure 7C). At the genus level, 600 mg/kg CGA increased the relative abundance of *Lactobacillus*, *Ruminococcus_torques_group*, and *norank_f__norank_o__Clostridia_UCG-014*, and decreased the relative abundance of *Rikenellaceae_RC9_gut_group*, *unclassified_f__Spirochaetaceae*, *Treponema*, *Erysipelatoclostridium*, *unclassified_f__Tannerellaceae*, *unclassified_f__Spirochaetaceae*, and *norank_f__F082*.

**Figure 8:**
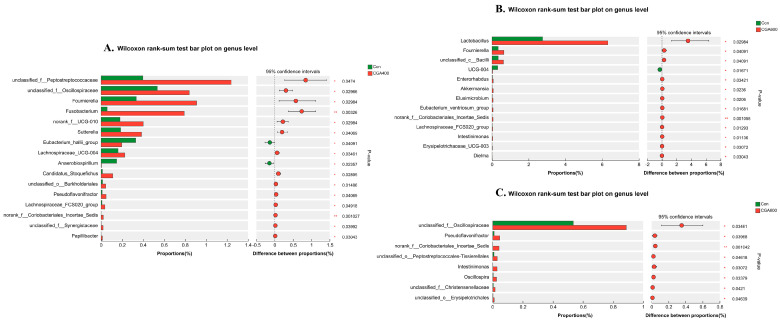
The species with differences in abundance between the control group and CGA addition groups ((**A**) 400 mg/kg CGA, (**B**) 600 mg/kg CGA, (**C**) 800 mg/kg CGA). *n* = 7 hens per group (at least 1 hen per replicate). Con: control group; CGA 400: 400 mg/kg CGA group; CGA 600: 600 mg/kg CGA group; CGA 800: 800 mg/kg CGA group. * *p*  < 0.05, ** *p*  < 0.01.

Further, we analyzed the species differences at the generic level; the results are shown in Figure 8. In comparison to the control group, 400 mg/kg CGA supplementation resulted in a notable rise in the relative abundance of beneficial microbes including *unclassified_f__Peptostreptococcaceae*, *unclassified_f_Oscillospiraceae*, *Fournierella*, *Lachnospiraceae_UCG-004*, *Candidatus_Stoquefichus*, *unclassified_o__Burkholderiales*, *Pseudoflavonifractor*, *Lachnospiraceae_FCS020_group*, *norank_f__Coriobacteriales_Incertae_Sedis*, and *Papillibacter*, and reduced the abundance of harmful microbes *Anaerobiospirillum* (*p* < 0.05). However, 400 mg/kg CGA treatment significantly enhanced cecum pathogenic bacteria abundances such as *Fusobacteriota* and *Sutterella* (*p* < 0.05) (Figure 8A). The relative abundance of beneficial bacteria including *Lactobacillus*, *Fournierella*, *unclassified_c__Bacilli*, *Akkermansia*, *Elusimicrobium*, *Eubacterium_ventriosum_group*, *norank_f__Coriobacteriales_Incertae_Sedis*, *Lachnospiraceae_FCS020_group*, and *Intestinimonas* were increased significantly in 600 mg/kg CGA supplementation group compared to control (*p* < 0.05) (Figure 8B). 800 mg/kg CGA significantly increased the abundance of beneficial microbes including *unclassified_f__Oscillospiraceae*, *Pseudoflavonifractor*, *norank_f__Coriobacteriales_Incertae_Sedis*, *Intestinimonas*, *Oscillospira*, and *unclassified_f__Christensenellaceae* (*p* < 0.05) (Figure 8C).

**Figure 9:**
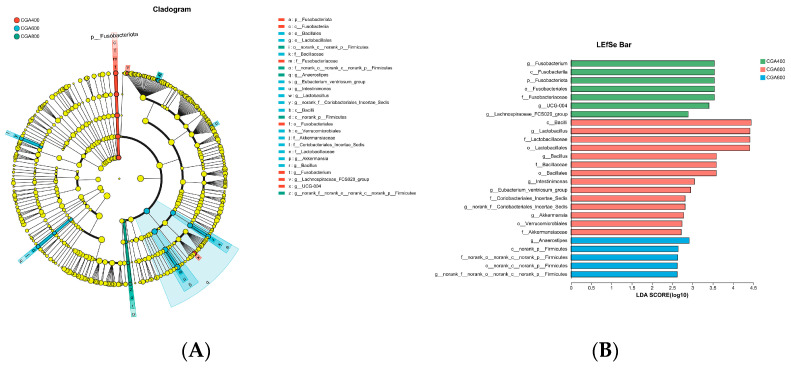
Taxonomic cladogram obtained from LEfSe analysis (**A**). Displayed are taxa exhibiting an LDA score exceeding 2.5 (**B**). *n* = 7 hens per group (at least 1 hen per replicate). CGA 400: 400 mg/kg CGA group; CGA 600: 600 mg/kg CGA group; CGA 800: 800 mg/kg CGA group.

Linear discriminant analysis (LDA) effect size (LEfSe) (LDA > 2.5) was utilized to indicate the difference in cecal microbiota in hens (Figure 9). The findings revealed significant enrichments in *Fusobacteriota* (from phylum to genus), *g_UCG-004*, and *g_ Lachnospiraceae_FCS020_group* in the 400 mg/kg CGA group. The 600 mg/kg CGA group exhibited significant enrichments in *Lactobacillales* (from order to genus), *Bacillales* (from order to genus), *Intestinimonas*, *Eubacterium_ventriosum_group*, *Coriobacteriales_Incertae_Sedis* (from family to genus), *Akkermansiaceae* (from family to genus), and *Verrucomicrobiales*. The 800 mg/kg CGA group showed significant enrichments in *Anaerostipes* and *norank_p__Firmicutes* (from class to genus).

## 4. Discussion

The persistent ovulation of laying hens during their peak reproductive period is hypothesized to induce oxidative stress, leading to intestinal disorders in chickens, including diarrhea and enteritis [28]. This is largely ascribed to the susceptibility of the intestinal epithelium, which serves as a defensive interface between the organism and its luminal surroundings, rendering it prone to oxidative damage from luminal oxidants [29]. Oxidative stress is characterized as a condition in which there is an imbalance in free radical generation and elimination [30]. Antioxidant enzymes are recognized for their crucial role in neutralizing free radicals [31]. In this research, CGA addition significantly reduced MDA and H_2_O_2_ levels in the ileum. Treatment with 600 mg/kg of CGA significantly enhanced the GSH-Px activity. However, no notable alterations in T-SOD and CAT activities were observed across all experimental groups. This observation is likely due to the hydroxyl groups present in CGA, which can neutralize free radicals, like superoxide anions and hydroxyl radicals, thereby safeguarding cells from oxidative injury [32].

The GI health significantly influences feed intake and the efficient absorption of nutrients [1,2]. The efficiency of nutrient digestion and absorption heavily depends on the morphological structure of intestinal villi. Typically, parameters such as intestinal VH, CD, VH/CD ratio, and goblet cell numbers are utilized to assess intestinal digestion and absorption capacity [33]. In general, the longer intestinal villi and reduced CD are related to improved intestinal structure and function, enhanced digestion and absorption capabilities, and increased disease resistance [34,35]. The work of Li et al. demonstrated that CGA treatment positively influenced the intestinal structure of the broiler, leading to a notable rise in ileal VH and VH/CD ratio [23]. In this research, CGA addition increased the intestinal VH and VH/CD ratio of laying hens. This suggests that CGA can improve the development of intestinal villi, potentially improving their intestinal digestion and absorption capacity, thereby enhancing egg production performance (egg production rate: 89.29 ± 0.32 (control), 91.25 ± 0.41 (400 mg/kg CGA), 91.48 ± 0.40 (600 mg/kg CGA), 94.09 ± 0.33 (800 mg/kg CGA); *p*-value < 0.001) in late-peak laying hens.

The gut barrier is a multifaceted structure that includes physical, chemical, immune, and microbial components [36]. The tight junction (TJ) complex, composed of claudin, occludins, zonula occludens, and junction adhesion molecules, is critical for maintaining gut barrier integrity by forming TJs among neighboring intestinal epithelial cells (IECs). These TJs serve to prevent the passage of pathogens while controlling the specific transfer of water, ions, and nutrients [37]. Studies have indicated that, under the conditions of stress, the connections between chicken IECs may become compromised, causing an elevated permeability of the intestinal barrier and subsequently stimulating the gut immune system by a greater influx of pathogens [38]. In this research, the findings demonstrated that CGA supplementation significantly upregulated ZO-1, Occludin, and Claudin1 gene expressions in the intestine of late-peak laying hens. This coordinates with the results reported by Chen et al., who observed a similar rise in mRNA levels of TJ proteins in the intestines of weaned piglets treated with CGA [39]. These results indicate that CGA addition can improve intestinal barrier integrity by enhancing the secretion of ZO-1, Occludin, and Claudin-1. Recent research has emphasized the beneficial role of IL-22 originating from group 3 innate lymphoid cells in preserving the intestinal barrier integrity [40]. IL-22 has been shown to enhance the intestinal barrier by upregulating TJ proteins [41], as well as strengthening mucosal defense through the induction of antimicrobial peptides and mucin production [42]. In addition, research has proved that IL-22 can also improve IEC proliferation by activating the STAT3 signaling pathway [43]. IL-22 regulation is primarily mediated by the AhR, a ligand-activated transcription factor crucial for maintaining intestinal barrier homeostasis [44]. Certain indole derivatives from the microbial breakdown of tryptophan are key endogenous AhR ligands [45]. Yu et al. demonstrated that CGA treatment caused a significant increase in metabolites related to tryptophan metabolism in mice’s cecum, including indole acetic acid and methyl indole-3-acetate [46]. In this research, CGA supplementation significantly upregulated AHR, IL-22, and STAT3 gene expression in the duodenum, jejunum, and/or ileum of laying hens. It can be inferred that CGA may enhance intestinal barrier function by improving the intestinal flora of laying hens, increasing metabolites related to tryptophan metabolism, and activating the AHR/IL-22/STAT3 pathway.

The GI is commonly acknowledged as the body’s largest immune organ, playing a critical role in maintaining immunological balance. The GI tract is estimated to accommodate about seventy percent of the body’s lymphocytes, solidifying its status as the primary immunological organ [3]. This investigation revealed a notable elevation in the serum and ileal IgG levels of layers following dietary supplementation with CGA. IgG is also known as IgY in birds. Its synthesis functions in complement activation and neutralization of various poisons in the immune reaction [47]. Meanwhile, the level of IgA gene expression in the ileum showed a similar trend. Prior research has shown that the secretory IgA prevents pathogenic bacteria from adsorbing and entering epithelial cells and protects IECs from intestinal poisons and pathogenic bacteria [48]. These results suggested that CGA may increase intestinal immunity to foreign stimuli by increasing immunoglobulin secretion. Further, we found that CGA has significantly upregulated the cluster of differentiation 3D (CD-3D) and CD4 expression in layers’ duodenum, jejunum, and ileum. Lymphocytes with distinct functions display different CD molecules. As an example, CD3 and CD4 are surface markers of total T lymphocytes and T helper cells, respectively [49]. CD3D is one of the components of the TCR/CD3 complex, and CD4 is an important immune cell surface molecule. Both of them participate in T cell activation and differentiation and are crucial in immune response regulation [50,51]. In the current study, the increased expression of the CD3D and CD4 genes in the intestines of laying hens fed with CGA indicated that the intestinal mucosal T cell immune system of laying hens fed CGA was better developed than that of the control. Additionally, various research has proven that CGA can suppress inflammation by blocking the phosphorylation of the NF-κB p65 component, leading to reduced levels of downstream inflammatory mediators such as interleukin IL-1 β [52]. However, in this research, 600 mg/kg CGA significantly reduced the expressions of IL-1 β gene, but did not change the level of NF-κB gene expression. Therefore, the results of CGA on the NF-κB pathway in the intestine of laying hens need to be further studied.

The intestinal flora is crucial for nutrient metabolism, gut development, and a fully functional immune system formation [4,5]. A study from Gonthier et al. showed that the bioavailability of CGA is largely dependent on its interaction with gut microbiota, which proved that CGA and intestinal flora had a close relationship [17]. The findings of this study indicated a notable difference in the structure of microbiota between the control and the diet added with 400 and 600 mg/kg CGA. The dominant phyla from each group were *Firmicutes* and *Bacteroidata*, while the dominant genera included Bacteroides, *Rikenellaceae_RC9_gut_group*, *unclassified_f_Lachnospiraceae*, *unclassidied_o_Bacteroidales*, *Ruminococcus_torques_group*, *Faecalibacterium*, and *Lactobacillus*. Notably, the relative abundance of *norank_f__Coriobacteriales_Incertae_Sedis* was significantly increased in all CGA supplementation groups compared to the control. This bacterium has been linked to the production of short-chain fatty acids (SCFAs) [53,54], which are recognized for their vital contribution to energy balance, colonic motility, immune modulation, and inflammation suppression [55]. In addition, we found that a variety of bacteria linked to the production of SCFAs were significantly enriched in different CGA addition groups. Specifically, *unclassified_f__Peptostreptococcaceae* was enriched in the 400 mg/kg CGA group, *unclassified_f_Oscillospiraceae* and *Pseudoflavonifractor* were enriched in the 400 and 800 mg/kg CGA groups, the Lachnospiraceae_FCS020_group was enriched in the 400 and 600 mg/kg CGA groups, *Oscillospira* was enriched in the 800 mg/kg CGA group, *Elusimicrobium* and *Eubacterium_ventriosum_group* were enriched in the 600 mg/kg CGA group, and *Intestinimonas* was enriched in the 600 and 800 mg/kg CGA groups. Eubacterium, a constituent of the core gut microbiome, is the predominant producer of butyrate in both gut and stool samples. Various species within the genus, such as *E. rectale*, *E. ramulus*, etc., are responsible for the synthesis of butyrate, a compound crucial for the maintenance of intestinal health [56,57]. *Lachnospiraceae* is a microbial group with a positive association with the contents of acetate and butyrate [58]. The members of the family *Peptostreptococcaceae* have been identified as early butyrate producers in samples obtained from 3-month-old infants [59]. *Intestinimonas* play a vital role in the transformation of lysine and fructose lysine into butyrate within the GI tract of humans [60]. Certain species of *Oscillospira* are likely capable of utilizing host glycans and potentially secreting the important SCFA butyrate [61,62]. *Elusimicrobium minutum*, the sole cultured representative in class *Elusimicrobia*, has been proven to produce acetate and alanine [63]. Some studies suggested that *Pseudoflavonifractor* is a probiotic whose main final metabolites are acetic acid and succinic acid [64,65]. In this investigation, a rise in the abundance of SCFA-producing bacteria in the gut of layers receiving CGA suggests that CGA could potentially enhance intestinal health by increasing intestinal SCFA production.

In addition to the above SCFA-producing bacteria, it was found that *Bacillus*, *Lactobacillus*, and *Akkermansia* were significantly enriched in the 600 mg/kg CGA group. *Bacillus* and *Lactobacillus* strains are important constituents of the gut microbiota and are commonly used as probiotics [66,67]. *Bacillus* strains are recognized for their efficacy in combating pathogenic bacteria, facilitating the proliferation of *Lactobacillus*, and preserving bacterial equilibrium [68]. Studies have demonstrated that probiotic *Bacillus* can enhance barrier function and immune response of IECs through modulating intestinal mucosa structure, TJs, and Toll-like receptor (TLR) pathways [69,70]. *Lactobacillus* species serve a vital microbial function by competitively displacing opportunistic pathogens within the gut environment, impeding pathogen adhesion to IECs, and directly killing pathogenic bacteria through generating lactic acid, acetic acid, propionic acid, and bacteriocins [71]. Apart from communicating with each other, these gut-dwelling *Lactobacillus* species also collaborate with the gut epithelium lining to maintain intestinal barrier integrity, promote mucosal defense, and boost host immune response [67]. Indole aldehydes, tryptophan metabolites generated by indigenous *L. reuteri* strains, stimulate the host AHR to enhance gut barrier function and antimicrobial defenses [72]. In addition to their microbial functions, commensal *Lactobacillus* species play a vital role in regulating both innate and adaptive immune responses through promoting T cells, Natural Killer cells, and macrophage differentiation, as well as stimulating cytokine production and TLRs. They can inhibit pathogenic bacteria adhesion to IECs by upregulating the expression of IgA-producing B cells in Peyer’s patches within the lamina propria [73]. The genus *Akkermansia* has been shown to enhance goblet cell numbers and reinforce barrier integrity [74,75]. Therefore, in this experiment, the enhancement in intestinal barrier function and immunity of laying hens fed CGA may be closely related to the enrichment of probiotic *Bacillus*, *Lactobacillus*, and *Akkermansia* in the gut, promoted by dietary supplementation of CGA.

## 5. Conclusions

Dietary CGA (600–800 mg/kg) effectively improved intestinal morphology, antioxidant status, barrier function, immune response, and beneficial microbiota growth in late-peak layers. This suggests that CGA supplementation can potentially improve intestinal digestion and absorption capacity.

## Figures and Tables

**Table 1:** Ingredient compositions and nutrient contents of basal diet for hens (as-fed basis).

Items	Value (%)	Nutrient Content ^§^	Value
Corn	61.85	Metabolism energy, MJ/Kg	11.07
Soybean meal (43% CP)	25.00	Crude protein, %	16.33
Fish meal (67% CP)	0.50	Ether extract, %	3.23
Soybean oil	0.50	Lysine, %	0.84
Limestone	7.00	Methionine, %	0.36
DL-Met (99.8%)	0.10	Methionine + cystine, %	0.68
L-Cys (99.0%)	0.05	Available phosphorus, %	0.32
Premix ^†^	5.00	Calcium, %	3.53
Total	100.00		

^†^ Premix: vitamin A: 8000 IU per kilogram of full price material; vitamin D3: 2400 IU; vitamin E: 12 IU; vitamin K3: 1.2 mg; vitamin B1: 1.82 mg; vitamin B2: 6.2.mg; vitamin B6: 2.5 mg; vitamin B12: 0.02 mg; D-biotin: 0.21 mg; niacin: 28 mg; folic acid: 0.88 mg; calcium D-pantothenate: 10 mg; choline chloride: 400 mg; calcium hydrogen phosphate: 12 mg; copper: 10 mg; manganese: 60 mg; iron: 60 mg; iodine: 0.20 mg; selenium: 0.5 mg. ^§^ Estimated from the Chinese feed database provided with tables of feed composition and nutritive values in China (2015).

**Table 2:** Primer used for quantitative real-time PCR.

Target Gene	Primer	Primer Sequence (5′-3′)	Accession No.	Product Size (bp)
β-actin	Forward	TCCCTGGAGAAGAGCTATGAA	NM_205518.1	113
	Reverse	CAGGACTCCATACCCAAGAAAG		
ZO-1	Forward	GATCTCCCTAAAGGCGAAGAAG	XM_046925209.1	415
	Reverse	GAACAGGCTGAGCAGAAAGA		
Claudin-1	Forward	GCTCACCAAAGAGGGAAGAA	NM_001013611.2	446
	Reverse	GTACAGGTCAGCATCAGATCAA		
Occludin	Forward	CTCTGCCTCATCTGCTTCTT	NM_205128.1	418
	Reverse	CATACTGGGACTCATCCAACTC		
Mucin-2	Forward	TTCATGATGCCTGCTCTTGTG	XM_040673077.2	93
	Reverse	CCTGAGCCTTGGTACATTCTTGT		
TLR4	Forward	GGAGTTGAGAGTGCTTCGTATT	NM_001030693.2	276
	Reverse	GGGTAGGTGCCATGATGAATTA		
Myd88	Forward	TCTGGTGACTGTGGAGCAAGGAA	NM_001030962.5	207
	Reverse	CCGCTTGTAGGAAGGCACTAATGG		
IL-1β	Forward	CTCTACATGTCGTGTGTGATGAG	NM_204524.2	249
	Reverse	CTTGTAGGTGGCGATGTTGA		
CD3D	Forward	TGCATCACTGGGCAAGATAA	NM_205512.2	250
	Reverse	CAGCAGCAAGTTCACAACAC		
CD4	Forward	CATTCCCAGCCCTTCAGTTT	NM_204649.2	218
	Reverse	CCAAGTACAGGTCCCATCTTTC		
NF-κB	Forward	CTCCTCAACCTCACTTCCTTAC	NM_001396396.1	205
	Reverse	GCTGTGTGCTTTACCTCTTTG		
IgA	Forward	ACCACGGCTCTGACTGTACC	S40610.1	100
	Reverse	CGATGGTCTCCTTCACATCA		
INF-γ	Forward	AAAGCCGCACATCAAACACA	NM_205149.2	64
	Reverse	GCCATCAGGAAGGTTGTTTTTC		
IL-22	Forward	CTGCTGTTGTTGCTGTTTCC	NM_001199614.1	231
	Reverse	CGGTTGTTCTCCCTGATGTT		
AHR	Forward	CTCATCTGGGTTTCTGGCTATG	XM_046910172.1	348
	Reverse	CTCTCACCCGTCTTCATCATTC		
STAT3	Forward	CACCACTGCTTTCCCTATTCT	NM_001398323.1	390
	Reverse	CTTCCTTTGTCCACCCTTCTT		

**Table 3:** Effects of CGA on antioxidant enzymes, MDA, and H_2_O_2_ concentrations in the ileum of laying hens ^1^.

Items	CGA ^2^ (mg/kg)				*p*-Value
	0 (Control)	400	600	800	
MDA ^2^, mmol/mgprot	5.36 ± 0.47 ^a^	2.71 ± 0.39 ^b^	3.40 ± 0.57 ^b^	2.07 ± 0.27 ^b^	0.001
GSH-Px ^2^, U/mgprot	278.44 ± 12.32 ^b^	246.08 ± 19.44 ^b^	377.90 ± 18.18 ^a^	251.26 ± 29.61 ^b^	0.001
T-SOD ^2^, U/mgprot	561.74 ± 23.05	536.25 ± 31.68	555.75 ± 47.70	542.92 ± 36.29	0.956
CAT ^2^, U/mgprot	12.82 ± 0.56	12.68 ± 1.24	12.50 ± 0.84	10.88 ± 0.74	0.387
H_2_O_2_^2^, mmol/gprot	7.50 ± 0.32 ^a^	5.18 ± 0.34 ^b^	5.14 ± 0.54 ^b^	5.59 ± 0.59 ^b^	0.005

^1^ Data are mean ± SEM (*n* = 12). Values with different superscript letters are significantly different (*p* < 0.05). ^2^ CGA: chlorogenic acid; CAT: catalase; GSH-Px: glutathione peroxide; T-SOD: total superoxide dismutase; MDA: malondialdehyde; H_2_O_2_: hydrogen peroxide.

**Table 4:** The effects of CGA supplementation on the intestinal morphology of laying hens ^1^.

Items	CGA ^2^ (mg/kg)				*p*-Value
	0 (Control)	400	600	800	
Duodenum					
VH ^2^, μm	1373.22 ± 44.48 ^c^	1551.08 ± 37.75 ^b^	1527.68 ± 15.78 ^b^	1716.70 ± 37.19 ^a^	<0.001
CD ^2^, μm	103.21 ± 5.60	88.53 ± 2.32	104.57 ± 4.60	97.01 ± 8.90	0.313
VH/CD ^2^	13.89 ± 1.10 ^b^	17.65 ± 0.80 ^ab^	14.82 ± 0.72 ^ab^	19.05 ± 1.68 ^a^	0.013
Jejunum					
VH, μm	1317.14 ± 38.59 ^b^	1217.49 ± 52.24 ^b^	1515.14 ± 16.57 ^a^	1198.42 ± 44.23 ^b^	<0.001
CD, μm	121.62 ± 6.89 ^a^	77.94 ± 5.74 ^b^	92.56 ± 4.04 ^b^	94.11 ± 5.05 ^b^	<0.001
VH/CD	11.04 ± 0.52 ^b^	16.49 ± 1.38 ^a^	16.62 ± 0.74 ^a^	13.20 ± 3.45 ^ab^	0.001
Ileum					
VH, μm	686.38 ± 22.33 ^c^	1027.15 ± 31.78 ^b^	1141.39 ± 30.23 ^a^	969.74 ± 28.72 ^b^	<0.001
CD, μm	99.56 ± 6.94	107.29 ± 4.20	93.73 ± 4.81	96.58 ± 4.59	0.311
VH/CD	7.23 ± 0.60 ^c^	9.76 ± 0.59 ^b^	12.50 ± 0.77 ^a^	10.73 ± 1.47 ^ab^	<0.001

^1^ Data are mean ± SEM (*n* = 12). Values with different superscript letters are significantly different (*p* < 0.05). ^2^ CGA: chlorogenic acid; VH: villus height; CD: crypt depth; VH/CD: villus height/crypt depth.

## Data Availability

The datasets are available in the Sequence Read Archive under the accession number PRJNA1142733 (Available online: https://www.ncbi.nlm.nih.gov/sra/PRJNA1142733, accessed on 1 August 2025).
